# Enhancement of CD8^+^ T-cell memory by removal of a vaccinia virus nuclear factor-*κ*B inhibitor

**DOI:** 10.1111/imm.12422

**Published:** 2015-04-14

**Authors:** Hongwei Ren, Brian J Ferguson, Carlos Maluquer de Motes, Rebecca P Sumner, Laura E R Harman, Geoffrey L Smith

**Affiliations:** Department of Pathology, University of CambridgeCambridge, UK

**Keywords:** CD8^+^ T-cell memory, *N1L* gene, nuclear factor-*κ*B inhibitor, vaccination, vaccinia virus

## Abstract

Factors influencing T-cell responses are important for vaccine development but are incompletely understood. Here, vaccinia virus (VACV) protein N1 is shown to impair the development of both effector and memory CD8^+^ T cells and this correlates with its inhibition of nuclear factor-*κ*B (NF-*κ*B) activation. Infection with VACVs that either have the *N1L* gene deleted (vΔN1) or contain a I6E mutation (vN1.I6E) that abrogates its inhibition of NF-*κ*B resulted in increased central and memory CD8^+^ T-cell populations, increased CD8^+^ T-cell cytotoxicity and lower virus titres after challenge. Furthermore, CD8^+^ memory T-cell function was increased following infection with vN1.I6E, with more interferon-*γ* production and greater protection against VACV infection following passive transfer to naive mice, compared with CD8^+^ T cells from mice infected with wild-type virus (vN1.WT). This demonstrates the importance of NF-*κ*B activation within infected cells for long-term CD8^+^ T-cell memory and vaccine efficacy. Further, it provides a rationale for deleting N1 from VACV vectors to enhance CD8^+^ T-cell immunogenicity, while simultaneously reducing virulence to improve vaccine safety.

## Introduction

Immunological memory provides protection against re-infection by pathogens encountered previously and in mammals is conferred by specific leucocyte populations that endure long after clearance of infection.^1^,^2^ Naive T-cell clones expand rapidly after T-cell receptor ligation and, although most die subsequently after clearance of the specific antigen, some survive to become long-lasting memory cells that protect against future infection.^3^,^4^ Induction of strong T-cell memory is desirable for vaccine development but factors that influence this are not fully understood. Two populations of memory T cells, called central and effector memory CD8^+^ and CD4^+^ T cells (T_CM_ and T_EM_, respectively), are defined by expression of specific surface markers. In mice these are CD62L and CD44 (T_CM_ being CD44^hi^ CD62L^hi^ and T_EM_ being CD44^hi^ CD62L^lo^) and are strongly induced by acute viral infections.^5^,^6^ These subsets are functionally distinct because T_CM_ cells mediate long-term protection, whereas T_EM_ cells provide immediate protection^6^ and they have distinct locations with T_CM_ being resident mainly in lymph nodes, whereas T_EM_ are predominantly in peripheral tissue. CD8^+^ T_CM_ and T_EM_ cells confer protection against several pathogens, although the T_CM_ subset may be more broadly protective.^7^–^10^

Vaccinia virus (VACV) was the live vaccine used to eradicate smallpox^11^ and induces long-lasting protective immunity,^12^–^15^ for review see ref. 16. Consequently, VACV is useful for studying the induction of immunological memory. Moreover, the engineering of VACV to express foreign antigens has made VACV a popular vector for vaccine development.^17^,^18^ VACV expresses about 200 proteins^19^ and many of these inhibit innate immunity.^20^ Studying such immunosuppressive proteins provides insight into how the innate immune system functions and may be suppressed,^21^–^23^ and their manipulation can improve VACV immunogenicity. For instance, deletion of genes encoding the chemokine binding protein A41,^24^ the interleukin-1*β* binding protein B15,^25^ or the interferon regulatory factor 3 inhibitor C6 enhanced immune responses.^26^,^27^ Such viruses are useful tools for studying how the innate immune response shapes adaptive immunity.^28^

This paper concerns VACV protein N1 and shows that its deletion or mutation can simultaneously reduce virus virulence and induce stronger CD8^+^ T-cell responses that confer enhanced protection against virus challenge. N1 is present in many, but not all, VACV strains and orthopoxviruses, for details see ref. 29, and is, for instance, present in VACV strain modified virus Ankara but is shortened from 117 to 113 amino acid residues by a frameshift mutation that removes the last 27 residues and replaces these with 23 unrelated residues.^30^ N1 is an intracellular homodimer expressed early during infection^29^ that inhibits activation of nuclear factor-*κ*B (NF-*κ*B),^31^–^33^ suppresses apoptosis^33^,^34^ and contributes to virus virulence.^29^,^33^,^35^ The crystal structure of N1 revealed a Bcl-2 fold^34^,^36^ and structure-based mutagenesis showed that inhibition of NF-*κ*B activation and apoptosis are separable.^33^ N1 mutants unable to block NF-*κ*B activation (I6E) or apoptosis (R58Y and Q61Y) were described and analysis of recombinant VACVs expressing these mutant N1 proteins showed that inhibition of NF-*κ*B activation, rather than apoptosis, was the predominant mechanism by which protein N1 contributed to VACV virulence.^33^ An additional mutant, R71Y, affected neither inhibition of apoptosis nor NF-*κ*B activation and a virus bearing this mutation has wild-type virulence.^33^

Here, these VACVs are used to study how NF-*κ*B activation during infection influences the development of cellular immunity, immunological memory and resistance to re-infection. Nuclear factor-*κ*B is crucial in regulating inflammation and cell proliferation, but there is little direct evidence of its role in development of immunological memory due to gross developmental defects in mice where NF-*κ*B signalling is suppressed. Recombinant VACVs with altered ability to suppress NF-*κ*B enable circumvention of this problem. Data presented show that intradermal (i.d.) infection with VACV lacking N1 (vΔN1) or bearing the I6E mutation (vN1.I6E) caused increased activation of CD8^+^ T cells compared with wild-type (WT) virus (vN1.WT), illustrating the importance of NF-*κ*B signalling for induction of T-cell responses. Furthermore, mice infected with vN1.I6E or vΔN1 developed increased numbers of CD8^+^ T_CM_ and T_EM_ cells that mediated enhanced protection against VACV challenge. This study illustrates how the innate immune response to viral infection driven by NF-*κ*B has a profound impact on the development of T-cell memory and provides a rationale for deleting the *N1L* gene, and possibly other inhibitors of NF-*κ*B, from VACV-based vaccines.

## Materials and methods

### Ethics statement

This work was conducted under licence PPL 70/7116 from the UK Home Office according to the Animals (Scientific Procedures) Act 1986.

### Mice and cell lines

Female C57BL/6 (B6) mice (Harlan) were housed under pathogen-free conditions. EL4 (H-2b) and P815 (H-2d) cells (both American Type Culture Collection, Manassas, VA) were cultured in RPMI-1640 (Gibco, Grand Island, NY) containing 10% fetal bovine serum (FBS) (Harlan Seralab, Haywards Heath, West Sussex, UK) and penicillin/streptomycin (50 μg/ml; Gibco).

### Viruses

Vaccinia virus strain WR recombinants vN1.WT and vΔN1^29^ and VACVs expressing N1 mutant protein I6E, R58Y, Q61Y and R71Y were described previously.^33^ Virus infectivity was titrated by plaque assay on BSC-1 cells.

### Murine infection models

Female C57BL/6 mice (6–8 weeks) were infected i.d. with 10^4^ plaque-forming units in both ear pinnae.^37^,^38^ Virus doses used to infect animals were always re-titrated to confirm the infectious dose administered. *In vivo* data shown are from one representative experiment, and all experiments were performed at least twice. To determine virus titres, infected ears were ground with a tissue homogenizer, subjected to three cycles of freezing and thawing and sonication, and the resulting homogenate was titrated on BSC-1 cells.^37^,^38^ To evaluate the degree of protection induced by i.d. infection, immunized mice were challenged by intranasal infection with the indicated dose of VACV strain WR as described.^39^

### Isolation of cell populations

Mice were killed and the liver, spleen, lung and lymph nodes were removed. Hepatic lymphocytes were prepared as described.^40^ Splenocytes and lymph node suspension cells were obtained by forcing the organ through a stainless steel mesh. Splenocytes were treated with 0·2% NaCl solution to remove erythrocytes. Lung pieces were incubated in RPMI-1640 with 5% FBS, 100 U/ml penicillin/streptomycin, 10 mm HEPES, 50 μm 2-mercaptoethanol, 20 mm l-glutamine containing 20 U/ml collagenase (Type Ia) and 1 μg/ml DNase (Type I) for 30 min before passing through a mesh. For preparation of cells for passive transfer to recipient mice, the mouse CD4^+^ or CD8^+^ T-cell isolation kit was used as indicated by the manufacturer (Miltenyi Biotec, Bergisch Gladbach, Germany) to deplete non-CD4^+^ or non-CD8^+^ cells on an autoMACS instrument.

### Antibodies, cell staining and flow cytometry

Anti-mouse CD3 (clone 145-2C11), CD4 (GK1.5), CD8 (5H10-1), B220 (RA3-6B2), NK1.1 (PK136), CD11b (M1/70), Ly-6G/Ly-6C (RB6-8C5), CD44 (IM7), CD62L (MEL-14), granzyme B (GB11), CD16/32 (2.4G2) and interferon-*γ* (XMG1.2) monoclonal antibodies (mAbs) were purchased from BD Biosciences (San Jose, CA) or Biolegend (San Diego, CA). The mAbs were purified or conjugated with FITC, Peridinin chlorophyll protein/cy5.5, allophycocyanin, phycoerythrin-Cy7, BV650 C or BV421. Isotype controls were used as negative controls. For intracellular staining, cells were incubated with Golgistop (BD Pharmingen, San Diego, CA) for 5 hr before analysis. After surface staining, samples were fixed, permeabilized using Cytofix/Cytoperm intracellular staining kit (BD Pharmingen), and incubated with the indicated mAb. Then cells were stained intracellularly for 30 min, washed and fixed in 1% paraformaldehyde (Sigma-Aldrich, St Louis, MO). Flow cytometry was performed with a BD LSR Fortessa (BD Biosciences), and data were analysed with FlowJo software (Tree Star Inc., Ashland, OR). LIVE/DEAD® Fixable Aqua Dead Cell Stain Kit (Life Technologies, Paisley, UK) was used to exclude non-viable cells from analysis. Flow cytometric gating strategies are shown in Supplementary Figure S3.

### DimerX assay to detect VACV specific CD8^+^ T cells

Recombinant soluble dimeric mouse H-2K^b^:Ig fusion proteins were purchased from BD Biosciences and the DimerX assay was performed according to the manufacturer's instructions. Briefly, 2 μg of H-2Kb:Ig fusion proteins were incubated overnight at 37° in PBS with a 40 m excess of B8_20_ peptide (TSYKFESV). Peptide-loaded dimers were then incubated for 1 hr at room temperature with phycoerythrin-coupled anti-mouse IgG1 (clone A85-1, BD Biosciences). Cells were labelled with DimerX and anti-CD8 (clone 53-6.7, BD Biosciences) for 1 hr on ice and washed twice before acquisition using a BD LSR Fortessa (BD Biosciences). Analysis was done using FlowJo software (Tree Star Inc.). Events were gated for live lymphocytes on FSC × SSC followed by CD8^+^ T cells × DimerX^+^ cells. Backgrounds as determined using irrelevant peptides were in the order of 0·5–0·8% and were subtracted from the values presented for test samples.

### ^51^Cr-release cytotoxic assay

Cytotoxic T lymphocyte activity was assayed by ^51^Cr-release assay.^24^ VACV-infected EL4 cells were used as targets for VACV-specific cytotoxic T lymphocyte lysis. In some experiments, CD8^+^ cells were depleted from liver and spleen cell suspensions by incubation with anti-CD8 mAb (clone 3.115) together with human complement. An isotype control mAb was used in parallel. Flow cytometry confirmed > 95% depletion of the desired cells. The remaining cells were used for cytotoxicity assays without adjustment for alteration in number during depletion. The cytotoxicity of purified natural killer (NK) cells was tested on VACV-infected P815 cells by ^51^Cr-release assay. The percentage of specific ^51^Cr-release was calculated as specific lysis = [(experimental release − spontaneous release)/(total detergent release − spontaneous release)] × 100. The spontaneous release values were always < 15% of total lysis.

### Cell depletion by antibody *in vivo*

Rat anti-CD8 (YTS169) or rat anti-CD4 (YTS 191.1) mAbs were concentrated from tissue culture supernatant by ammonium sulphate precipitation and quantified by ELISA. Depleting antibodies (0·3 mg) were injected into the peritoneal cavity of naive recipient mice 10, 8 and 6 days before transfer of 10^6^ CD8^+^ or CD4^+^ T cells from immunized or naive mice. The depletion of specific T-cell populations was analysed by FACS and showed that > 95% of specific cells were depleted.

### Serum antibody titration

To measure the neutralizing titre of anti-VACV antibodies, vaccinated mice were exsanguinated at 28 days post-infection (p.i.), and sera were prepared and heated at 56° for 30 min to inactivate complement. Twofold dilutions of sera in Dulbecco's modified Eagle's medium (Gibco) supplemented with 2% FBS were prepared and were incubated with VACV intracellular mature virus (purified by sucrose density gradient centrifugation) for 1 hr at 37° before plaque assay on BS-C-1 cells. ND_50_ (Neutralisation dose 50) values represent the reciprocal of the serum dilution giving 50% reduction in plaque number compared with virus incubated without serum.

### Statistical analyses

Data were analysed using GraphPad Prism 5 software (GraphPad Software Inc., La Jolla, CA), represented as mean with the standard error of the mean (SEM), and assessed for significance using the Mann–Whitney *U* or Student's *t*-test statistics. *P*-values < 0·05 were considered statistically significant. *P < 0·05, ** *P* < 0·01.

## Results

### Deletion of VACV N1 increases effector CD8^+^ T-cell numbers during acute infection

**Figure 1:**
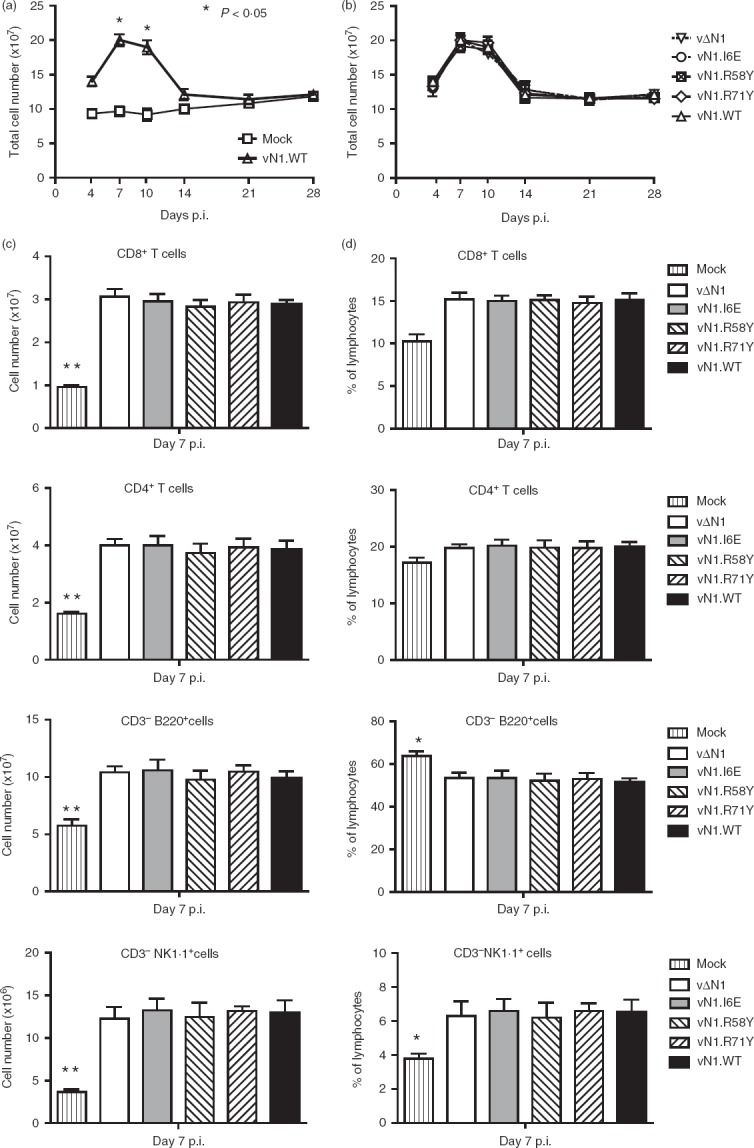
N1 mutation does not affect splenic lymphocyte numbers following infection. Groups of five mice were infected intradermally with the indicated vaccinia viruses (VACVs) and splenic lymphocyte populations were counted at 7, 14, 21 and 28 days post-infection. (a, b) Total cell numbers. (c, d) T-cell and B-cell populations presented as total cell number (c) and as percentages of total lymphocytes (d). Results are expressed as mean ± SEM. Statistical comparison of cells from mock-infected mice with virus-infected mice: *P <* *0·05, **P <* *0·01.

Intradermal infection with vN1.WT, vΔN1 or viruses with single amino acid mutations in the N1 protein did not affect virus replication *in vivo* early (2 days) p.i. (see Supporting information, Fig. S1), as noted earlier for vN1.WT, vΔN1 or revertant viruses.^29^ To investigate if blocking NF-*κ*B or apoptosis affected virus immunogenicity, mice were immunized i.d. with these viruses to mimic dermal vaccination and splenic T cells were analysed 1 month thereafter. Total splenic cells increased substantially early after infection (being maximal 7–10 days p.i.), but there were no differences between vN1.WT and the N1 mutant viruses in the magnitude or kinetics of this response (Fig.1a, b). The absolute numbers of T cells (CD3^+^ CD4^+^, CD3^+^ CD8^+^), B cells (CD3^−^ B220^+^) and NK cells (CD3^−^ NK1.1^+^) in the spleen at 7 days p.i. increased after infection (Fig.1c), but no differences were observed between the viruses. The proportion of splenic T cells (expressed as a percentage of total lymphocytes) increased after infection, whereas this value decreased for B cells (Fig.1d). The proportions of splenic macrophages (CD11b^+^ Ly6G^−^) and neutrophils (CD11b^+^ Ly6G^+^) (not shown) were similar to mock infection for all viruses. Analysis of cells in the posterior cervical lymph nodes proximal to the infection site showed similar results for the total cell numbers (see Supporting information, Fig. S2a), CD8^+^ T cells (Fig. S2b) and other cell subsets including CD4^+^ T cells, B cells, NK cells, macrophages and neutrophils (not shown).

**Figure 2:**
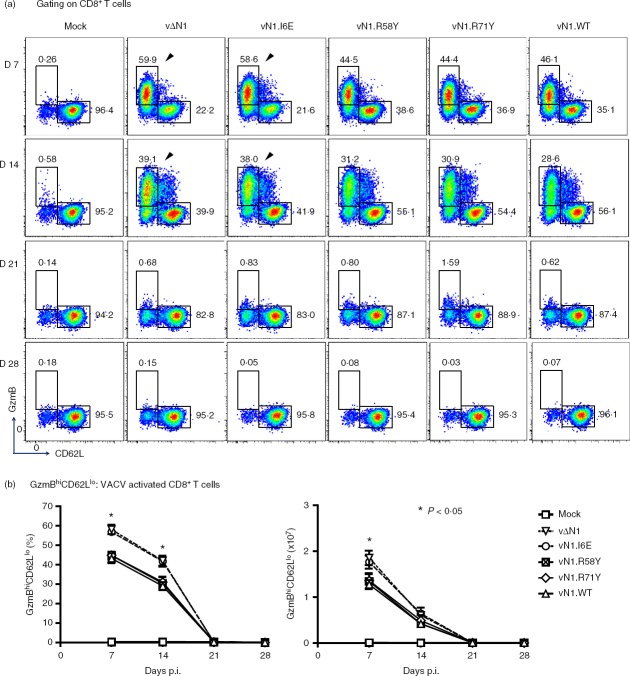
Infection with vΔN1 or vN1.I6E induces enhanced numbers of effector CD8^+^ T cells. Mice were infected intradermally with the indicated vaccinia viruses (VACVs) and populations of splenic GzmB^hi^ CD62L^lo^ CD8^+^ T cells were counted at 7, 14, 21 and 28 days post-infection. (a) Flow cytometry scatter plots from representative samples from individual mice. The arrows emphasize the greater percentage of GzmB^hi^ CD62L^lo^ CD8^+^ T cells following infection with vN1.I6E or vΔN1 compared with other viruses. (b) Graphs showing the proportion of total CD8^+^ T cells that were GzmB^hi^ CD62L^lo^ (left) and the absolute numbers of GzmB^hi^ CD62L^lo^ cells (right panel) (mean ± SEM). *P <* *0·05, *n* = 5.

**Figure 3:**
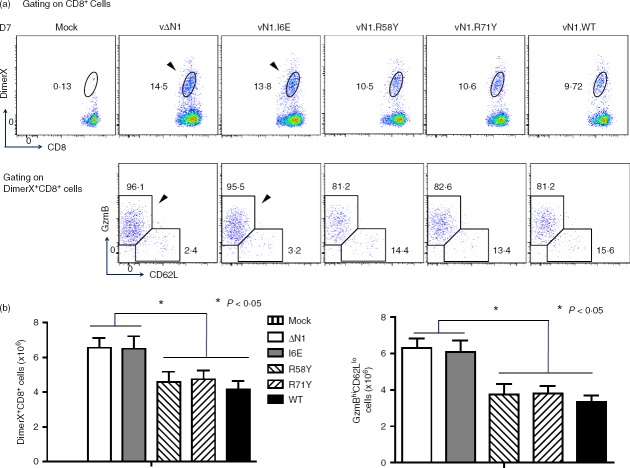
Infection with vΔN1 or vN1.I6E induces enhanced numbers of vaccinia virus (VACV) -specific effector CD8^+^ T cells. Mice were infected intradermally with the indicated VACVs and populations of splenic DimerX^+^ CD8^+^, and GzmB^hi^ CD62L^lo^ of DimerX^+^ CD8^+^ T cells were counted at 7 days post-infection. (a) Flow cytometry scatter plots from representative samples from individual mice. The arrows emphasize the greater percentage of DimerX^+^ CD8^+^ and GzmB^hi^ CD62L^lo^ of Dimer^+^ CD8^+^ T cells following infection with vN1.I6E or vΔN1 compared with other viruses. (b) Graphs showing the absolute numbers of DimerX^+^ CD8^+^ T cells (left) and of GzmB^hi^ CD62L^lo^ cells (right panel) (mean ± SEM). *, *P *<* *0·05, *n* = 5.

**Figure 4:**
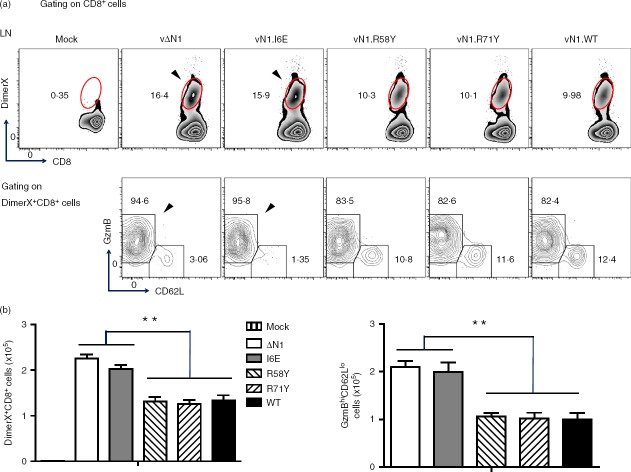
Infection with vΔN1 or vN1.I6E induces enhanced numbers of effector CD8^+^ T cells in the vaccinia virus (VACV) -specific CD8^+^ T cells in the draining lymph nodes. Mice were infected intradermally with the indicated VACVs and populations of DimerX^+^ CD8^+^, and GzmB^hi^ CD62L^lo^ of DimerX^+^ CD8^+^ T cells in the draining lymph nodes were counted at 7 days post-infection. (a) Flow cytometry scatter plots from representative samples from individual mice. The arrows emphasize the greater percentage of DimerX^+^ CD8^+^ and GzmB^hi^ CD62L^lo^ of DimerX^+^ CD8^+^ T cells following infection with vN1.I6E or vΔN1 compared with other viruses. (b) Graphs showing the absolute numbers of DimerX^+^ CD8^+^ T cells (left) and GzmB^hi^ CD62L^lo^ of DimerX^+^ CD8^+^ T cells (right panel) (mean ± SEM). **P < 0·01, *n* = 5.

During acute VACV infection, activated CD8^+^ T cells have high granzyme B (GzmB) and low CD62L expression (GzmB^hi^ CD62L^lo^) and this population can be detected without the requirement for *ex vivo* peptide antigen stimulation.^41^ These cells were analysed at different times p.i and in naive mice, ∼ 95% of splenic CD8^+^ T cells were in the resting, GzmB^lo^ CD62L^hi^, population and < 1% were GzmB^hi^ CD62L^lo^. However, 7 days p.i. with vN1.WT, vN1.R58Y or vN1.R71Y approximately 45% of splenic CD8^+^ T cells were GzmB^hi^ CD62L^lo^. Notably, after infection with vΔN1 or vN1.I6E this population increased to > 60% and the differences between these viruses and vN1.WT, vN1·58Y and vN1.R71Y were statistically significant (Fig.2a, b). Similar results were obtained 14 days p.i. when vΔN1 and vN1.I6E caused an increased proportion of activated CD8^+^ T cells. By days 21 and 28 p.i. the activated CD8^+^ T-cell population had decreased to resting levels in all groups (Fig.2). In conclusion, deletion of N1 or I6E mutation increased the number of activated CD8^+^ splenocytes (Fig.2b). To quantify VACV-specific CD8^+^ T cells, DimerX reagent loaded with the immunodominant VACV B8_20–27_ peptide was used. Consistent with a previous study,^41^ VACV-specific GzmB^hi^ CD62L^lo^ CD8^+^ T cells were identified in the spleen at day 7 p.i., and their proportion and absolute number increased upon deletion of N1 or I6E mutation (Fig.3a, b). In the draining lymph nodes this difference was more pronounced (*P* < 0·01) (Fig.4). Therefore, either removal of N1, or its mutation to ablate inhibition of NF-*κ*B, induced greater numbers of CD8^+^ effector T cells following infection.

### vΔN1 induces enhanced development of immunological memory

**Figure 5:**
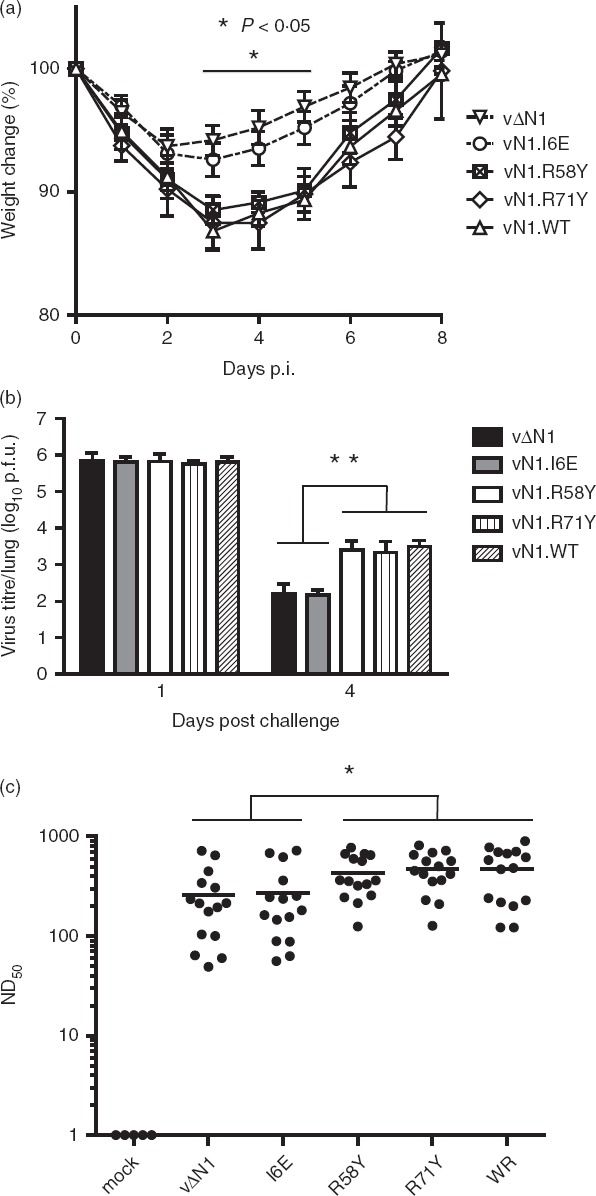
Vaccinia virus (VACV) lacking N1 or expressing N1.I6E induce better protection to virus challenge. (a) Groups of five mice were infected intradermally with the indicated viruses. At 28 days post-infection mice were challenged intranasally with VACV (5 × 10^6^ plaque-forming units of VACV WR) and weight change was monitored. Each mouse was compared to its original weight on day zero and data are expressed as the percentage ± SEM. (b) Groups of five mice were infected and challenged as (a), killed on day 1 or day 4 post-challenge (p.c.) and virus titres in the lungs were measured by plaque assay. Data are mean titre ± SEM, **P <* *0·01. (c) Sera from mice infected as in (a) were collected 28 days post-infection and assayed for neutralization of VACV strain WR. The median value for each population is represented by a horizontal black bar. Significant differences between groups are shown, Mann–Whitney *U*-test. *P <* *0·05, *n* = 15.

To test if changes induced by N1 mutation influenced protection against re-infection, mice were immunized i.d. with WT or mutant viruses and challenged intranasally 4 weeks later (when activated CD8^+^ T cells had returned to resting levels) with a dose of VACV WR (5 × 10^6^ plaque-forming units) representing > 200 LD_50_ for naive mice.^29^,^42^ Mice vaccinated with vΔN1 or vN1.I6E showed better protection against challenge, characterized by reduced weight loss and quicker recovery, compared with those immunized with vN1.WT, vN1.R58Y or vN1.R71Y (Fig.5a). Also, the virus titre in lungs 4 days after virus challenge was lower following immunization with vΔN1 or vN1.I6E compared with other groups (Fig.5b). No virus was detected in spleen after challenge for any of the virus groups (data not shown). Collectively, although deletion or I6E mutation of N1 reduced virulence, these changes enhanced immunological memory following vaccination.

To understand the basis of enhanced protection, VACV-specific antibodies were measured at 28 days p.i by plaque reduction neutralization assay.^43^ This showed that all groups of immunized mice had high serum antibody titres, but titres induced by immunization with vΔN1 or vN1.I6E were lower (*P* < 0·05) than mice infected with vN1.WT or vN1.R58Y and vN1.R71Y (Fig.5c). Therefore, antibody responses did not explain the enhanced protection induced by vΔN1 and vN1.I6E, suggesting that cellular immunity might be responsible.

### Deletion of VACV N1 results in enhanced CD8^+^ T-cell effector functions

**Figure 6:**
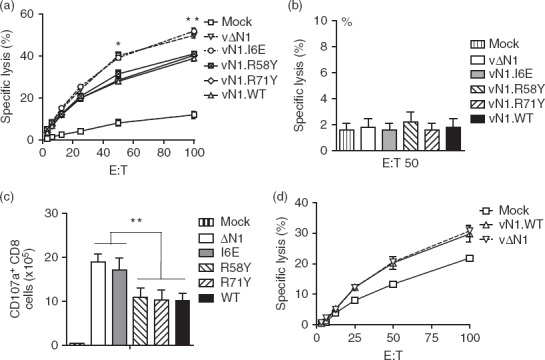
Deletion or I6E mutation of N1 enhances CD8^+^ T-cell cytotoxicity. (a) Mice were infected intradermally with the indicated viruses and splenic lymphocytes were harvested at 28 days post-infection and tested for their ability to lyse vaccinia virus (VACV) -infected EL4 cells by chromium release assay. Data are presented as percentage cell lysis at various effector to target (E : T) cell ratios. *P <* *0·05, **P <* *0·01, *n* = 5. (b) Lymphocytes were prepared as in (a) and pre-incubated with a CD8 blocking monoclonal antibody before cytotoxic activity was assayed as in (a). (c) Lymphocytes were prepared as in (a). Histograms showing the absolute number of CD8^+^ cells expressing CD107a following stimulation with VACV B8 peptide. (d) Cytotoxicity assay as in (a) but with purified splenic natural killer cells and using VACV-infected P815 cells as targets. Results are expressed as mean ± SEM.

To investigate T-cell effector functions, the killing activity of CD8^+^ T cells was assessed 28 days p.i with vN1.WT or the mutant N1 viruses by *ex vivo* cytotoxicity assay (Fig.6a). Splenic lymphocytes from mice immunized with vΔN1 or vN1.I6E showed significantly higher cytotoxicity against VACV-infected autologous target cells compared with cells derived from mice immunized with vN1.WT, vN1.R58Y or vN1.R71Y (Fig.6a). Notably, differences between groups and the cytotoxicity of lymphocytes were abolished by CD8^+^ T-cell depletion with specific mAb (Fig.6b). Analysis of hepatic lymphocytes gave similar results (not shown). Consistent with their enhanced killing activity, splenic CD8^+^ T cells expressed significantly greater CD107a at 28 days p.i. with vΔN1 or vN1.I6E (*P* < 0·01) than with vN1.WT, vN1.R58Y or vN1.R71Y (Fig.6c). Natural killer cell responses were not responsible for the enhanced protection because, although splenic NK cells from immunized mice lysed VACV-infected targets better than mock-infected cells, there were no differences between mice immunized with vN1.WT or vΔN1 (Fig.6d).

### vΔN1 and vN1.I6E induce increased populations of CD8^+^ T_CM_ and T_EM_ cells

**Figure 7:**
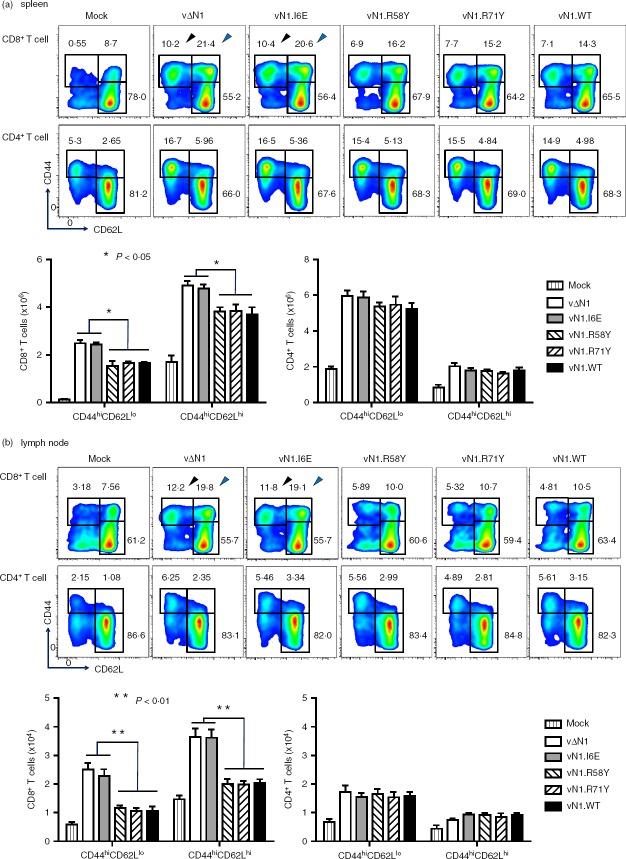
Deletion or I6E mutation of N1 enhances CD8^+^ central and effector memory T-cell (T_CM_ and T_EM_) populations. Mice were infected intradermally with the indicated viruses and 28 days later populations of CD8^+^ or CD4^+^ CD44^hi^ CD62L^lo^ (T_EM_) and CD44^hi^ CD62L^hi^ (T_CM_) cells were counted from (a) the spleen or (b) the draining lymph nodes. These data are presented as scatter plots with arrows indicating the gates corresponding to CD44^hi^ CD62L^lo^ populations (black arrow) or CD44^hi^ CD62L^hi^ (blue arrow, top panels), or as histograms showing absolute numbers of the specific populations as a proportion of total CD8^+^ or CD4^+^ cells. *P <* *0·05, **P < 0·001, *n* = 5. Results are expressed as mean ± SEM.

**Figure 8:**
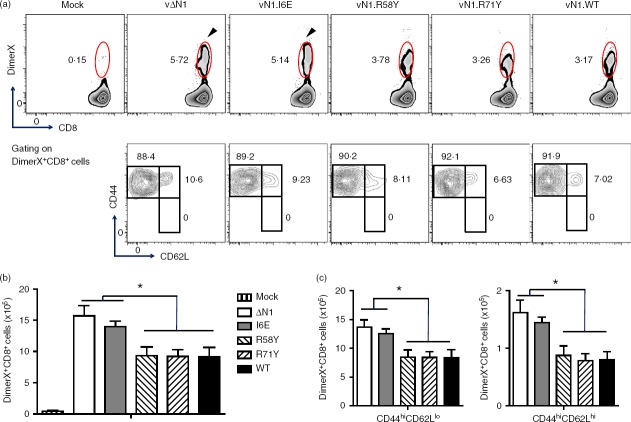
Deletion or I6E mutation of N1 enhances vaccinia virus (VACV) -specific CD8^+^ central and effector memory T-cell (T_CM_ and T_EM_) populations. Mice were infected intradermally with the indicated VACVs and populations of splenic lymphocytes that were DimerX^+^ CD8^+^, and T_EM_ or T_CM_ of DimerX^+^ CD8^+^ T cells, were counted at 28 days post-infection. (a) Flow cytometry scatter plots from representative samples from individual mice. The arrows emphasize the greater percentage of DimerX^+^ CD8^+^ following infection with vN1.I6E or vΔN1 compared with other viruses. (b) Graphs showing the absolute numbers of DimerX^+^ CD8^+^ T cells. (c) Graphs showing the absolute numbers of T_EM_ and T_CM_ of DimerX^+^ CD8^+^ T cells (mean ± SEM). *P < 0·05; *n* = 5.

The enhanced cytotoxicity of CD8^+^ T cells 28 days p.i. with vΔN1 and vN1.I6E indicated phenotypic differences between these cells and those from mice infected with the other viruses despite activation markers having returned to baseline by day 21 p.i. (Fig.2). Therefore, CD8^+^ T_CM_ and T_EM_ cells in the spleen and draining lymph node were analysed 28 days after i.d. infection. After vN1.I6E infection 10·4 ± 1·5% of splenic CD8^+^ T cells were CD44^hi^ CD62L^lo^ (T_EM_) and 20·6 ± 1·9% were CD44^hi^ CD62L^hi^ (T_CM_) and similar results were obtained with vΔN1. However, only 7·1 ± 1·3% CD8^+^ T_EM_ and 14·7 ± 2·2% of CD8^+^ T_CM_ cells were induced by vN1.WT, vN1.R58Y or vN1.R71Y and the differences between these groups and the vN1.I6E/vΔN1 groups were statistically significant (*P* < 0·05) (Fig.7a). In the draining lymph nodes this enhancement was even more pronounced (*P* < 0·01) (Fig.7b). In contrast, CD4^+^ T_CM_ and T_EM_ populations were indistinguishable between viruses (Fig.7a, b). Analysis by DimerX staining demonstrated a higher proportion and absolute number of VACV-specific CD8^+^ T (DimerX^+^CD8^+^) cells at 28 days p.i. with vΔN1 and vN1.I6E compared with vN1.WT, vN1.R58Y or vN1.R71Y (*P* < 0·05) (Fig.8a, b). In this population there were no naive T cells (CD44^lo^ CD62L^hi^) and only T_EM_ and T_CM_ remained (Fig.8a lower panels). Infection with vΔN1 or vN1.I6E induced greater numbers of T_EM_ and T_CM_ cells than vN1.WT, vN1.R58Y or vN1.R71Y (*P* < 0·05) (Fig.8c). In conclusion, N1 reduces development of CD8^+^ T-cell central and effector memory and this correlates with inhibition of NF-*κ*B.

### CD8^+^ T-cell effector functions are enhanced after challenge of vΔN1- or vN1.I6E-vaccinated mice

**Figure 9:**
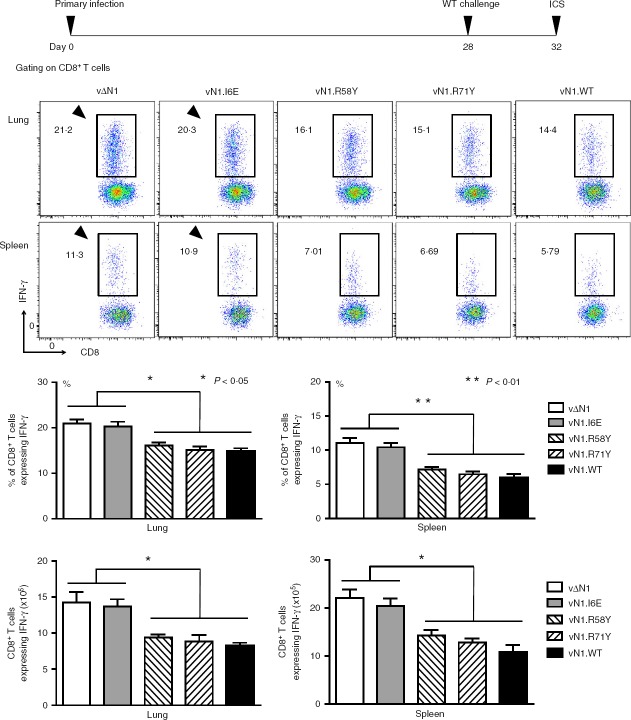
Deletion or I6E mutation of vaccinia virus (VACV) N1 enhances the effector function of memory CD8^+^ T cells. Mice were infected intradermally with the indicated viruses and challenged intranasally 28 days post-infection with 5 × 10^6^ plaque-forming units of VACV WR. Lung or splenic lymphocytes were isolated 4 days later and the CD8^+^ T-cell populations were assayed by intracellular cytokine staining (ICS) for interferon-*γ* (IFN-*γ*) production. Data are presented as scatter plots (top panels) and as histograms indicating the percentage of IFN-*γ*^+^ CD8^+^ T cells (middle panels), or the absolute cell number of IFN-*γ*^+^ CD8^+^ T cells (bottom panels). *P <* *0·05, **P < 0·001, *n* = 5. Results are expressed as mean ± SEM.

To address if enhanced CD8^+^ T-cell memory influences their effector function, interferon-*γ* production by these cells was investigated 4 days after re-infection. Cells from lungs and spleen were stimulated with peptides from VACV protein B8 (the interferon-*γ* receptor^44^) that are recognized by MHC class I restricted CD8^+^ T cells^45^ and interferon-*γ* production was quantified by intracellular cytokine staining. Interferon-*γ* production by cells from both organs was higher following vΔN1 and vN1.I6E infection compared with other viruses (Fig.9). Similar data were obtained with CD8^+^ T cells from the draining lymph nodes (data not shown). Therefore, CD8^+^ T-cell memory induced by vΔN1 or vN1.I6E infection correlates with enhanced effector function of these cells in response to secondary infection.

### CD8^+^ T cells from vN1.I6E-vaccinated mice confer enhanced protection

**Figure 10:**
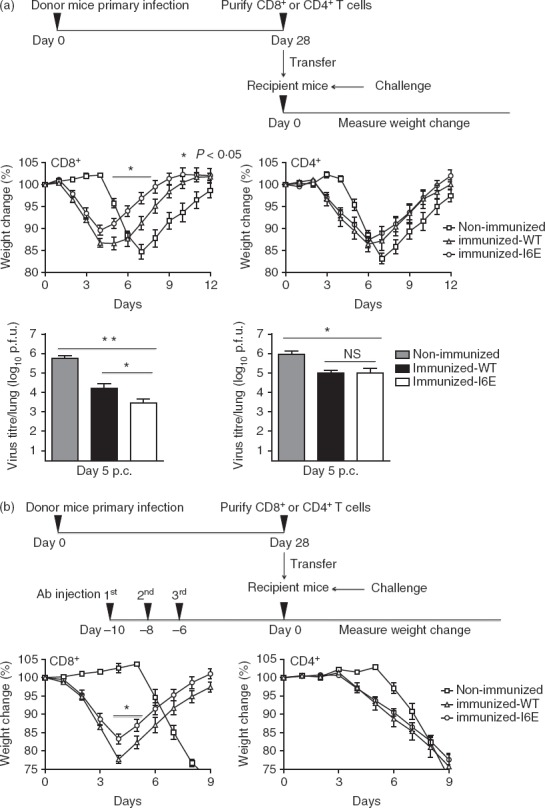
CD8^+^ T cells from vN1.I6E-infected mice confer enhanced protection. (a) Mice were infected intradermally with vN1.WT or vN1.I6E or mock infected and either CD8^+^ or CD4^+^ cells were isolated 28 days post-infection and 10^6^ cells were transferred into naive recipient mice. The recipient mice were challenged 6 hr later with 3 × 10^3^ plaque-forming units. of vaccinia virus (VACV) WR and weight change (middle panels) was monitored. *P <* *0·05, *n* = 5. Lower panels show virus titres in the lungs 5 days post-challenge. Data are mean titre ± SEM, *P <* *0·05, **P <* *0·01. NS = non-significant. (b) As (a), except the naive recipient mice were depleted for CD8^+^ or CD4^+^ T cells by administration of monoclonal antibody at 10, 8 and 6 days before transfer of cells. CD4^+^ T cells were transferred to mice depleted of CD4^+^ T cells, or CD8^+^ T cells were transferred to mice depleted of CD8^+^ T cells. *P <* *0·05, *n* = 5. Results are expressed as mean ± SEM.

Finally, the ability of T-cell subsets to confer protection against challenge with VACV was examined by passive transfer. Splenic CD8^+^ and CD4^+^ T cells were purified from naive or vaccinated mice 28 days p.i. and transferred to naive mice that were challenged with VACV. Mice infused with an equivalent number of additional naive CD8^+^ or CD4^+^ T cells were equally susceptible to subsequent virus challenge (data not shown), showing that more naive cells *per se* did not influence outcome. However, mice that received CD8^+^ or CD4^+^ T cells from vN1.WT immunized mice responded differently to challenge. First, these animals lost weight sooner than mice receiving naive cells (Fig.10). This effect has been observed repeatedly following intranasal challenge of VACV immunized mice and was attributable to lung immune pathology.^24^–^26^ Passive transfer of anti-VACV antibody before challenge did not enhance disease symptoms,^46^ suggesting that the effect was via cellular immunity. Figure10 shows that this phenomenon is mediated by T cells and that CD8^+^ cells play a greater role than CD4^+^ cells. Second, mice receiving either CD8^+^ or CD4^+^ T cells from immunized mice were protected better than those that received equivalent cells from naive mice, and this was characterized by a lower weight loss, lower virus titres and more rapid recovery (Fig.10a). Notably, the transfer of CD8^+^ T cells from mice immunized with vN1.I6E enhanced this protection, and reduced virus titres further, while the transfer of CD4^+^ T cells from these mice conferred no additional benefit over those from vN1.WT immunized mice (Fig.10a).

This beneficial effect of CD8^+^ T cells from vN1.I6E immunized mice was also seen if the recipient mice were depleted of CD8^+^ T cells by addition of mAb before passive transfer (Fig.10b). Flow cytometry showed that this treatment had depleted > 95% of CD8^+^ T cells at the time of challenge, and the mAb had declined to levels unable to affect the function of infused CD8^+^ T cells upon transfer (A Cooke, personal communication). As above, CD8^+^ T cells from vN1.I6E immunized mice conferred greater protection than cells from vN1.WT immunized mice, but the N1.I6E mutation did not influence protection from CD4^+^ cells (Fig.10a, b). Therefore, CD8^+^ and CD4^+^ memory T-cell populations confer protection against VACV challenge and the I6E mutation enhances protection from the CD8^+^ population. Hence, the inhibition of NF-*κ*B by N1 correlates with a profound and specific effect on the generation of CD8^+^ T-cell memory.

## Discussion

Despite encoding scores of immunomodulatory proteins, deletion of individual VACV genes can impact on virulence and immunogenicity.^20^ Previous studies showed that VACV protein N1, or its ectromelia virus counterpart, contributed to virulence in several infection models,^29^,^35^,^47^–^49^ affected T-cell and NK cell responses during primary infection^47^,^48^ and an N1 deletion mutant induced better protection from challenge.^49^ However, it was unknown if these effects of N1 were attributable to inhibition of apoptosis or NF-*κ*B or both activities, whether this phenotype was evident following immunization by dermal vaccination and challenge via a heterologous route, and what mechanism mediated the enhanced protection. Subsequently, using VACV N1 mutants that discriminate between inhibition of apoptosis and NF-*κ*B, it was shown that virulence correlated with inhibition of NF-*κ*B.^33^ Here, these mutants were used to investigate the consequence of inhibiting NF-*κ*B on the development of adaptive immunity using i.d. infection, mimicking dermal vaccination. N1 is shown to hinder the development of CD8^+^ T-cell effector and memory functions and this correlates with its ability to block NF-*κ*B activation.

During primary infection with VACV, deletion of N1 or a I6E mutation resulted in enhanced CD8^+^ T-cell (but not CD4^+^ or NK) cytotoxicity, increased numbers of CD8^+^ (but not CD4^+^) T_CM_ and T_EM_ cells, and better protection against challenge with VACV. In contrast, mutation of the BH3 binding groove of N1, which obviated the ability of N1 to bind Bad and Bid and to disrupt apoptotic signalling,^33^ did not affect T-cell responses. Therefore, N1 influences immunological memory, specifically CD8^+^ T cells, and this correlates with inhibition of NF-*κ*B signalling. Passive transfer demonstrated that CD8^+^ and CD4^+^ T cells from immunized mice provided protection against VACV challenge, but CD8^+^ T cells transferred from vN1.I6E-immunized mice conferred enhanced protection compared with CD8^+^ T cells from vN1.WT-immunized mice. Therefore, enhancement of the CD8^+^ T-cell response correlates with loss of NF-*κ*B inhibition.

During acute virus infection the inflammatory environment influences the proliferation and development of effector and memory T-cell populations. Inflammation is the third signal required for optimal T-cell activation, where antigen stimulation and co-stimulation comprise signals one and two, respectively.^50^,^51^ Interleukin-2, interleukin-12 and interferons influence the differentiation of naive T-cell precursors into their effector and memory populations and other cytokines may also contribute.^50^,^52^ Co-stimulation is provided mainly by 4-1BB and CD27, although CD40, CD28 and OX40 can also contribute to signal 2.^5^,^50^ Following clearance of antigen, interleukin-15 and interleukin-7 drive the proliferation and maintenance of the CD8^+^ memory populations and anti-apoptotic factors, such as Bcl_XL,_ are essential to avoid activation-induced death of these cells.^5^,^53^ Here we show that modulation of NF-*κ*B signalling by VACV protein N1 during primary infection affects the effector and memory CD8^+^ T-cell response, and this is consistent with NF-*κ*B regulating the expression of many cytokines, co-stimulatory molecules and maintenance factors needed for CD8^+^ T-cell responses.^54^ Transgenic mouse models for studying the impact of NF-*κ*B on these processes are hampered by the profound effects on the development of haematopoietic cells and/or systemic hyper-inflammation deriving from deletion or inhibition of NF-*κ*B.^55^–^57^ Hence, using mutant viruses to modify specific signalling pathways during infection provides an alternative way to assess the effects of NF-*κ*B, or other inflammatory responses, on immunological memory development in a wild-type host. In addition, data produced in this study showed an unexpected specificity for enhancing CD8^+^ T-cell function without modulating CD4^+^ T-cell function to the same degree by direct modification of NF-*κ*B during acute infection. In the future, mutant VACVs will allow aspects of the mechanisms of CD8^+^ and CD4^+^ T-cell responses during acute virus infection to be dissected.

The impact of innate immunity on the development of immunological memory is of great interest for vaccine development. Adjuvants help memory development^28^,^58^ by activating pattern recognition receptors and thereby transcription factors, including NF-*κ*B, which induce a favourable inflammatory cytokine environment at the site of antigen exposure during vaccination. The activity of N1 during VACV infection, namely blocking NF-*κ*B and inhibiting CD8^+^ T-cell memory, is opposite to the activity of a vaccine adjuvant. Therefore, deleting N1 from VACV is a logical strategy to improve immunogenicity, especially if cytotoxic T-cell activity is required. Indeed, removal of VACV immunomodulators can enhance memory responses during vaccination.^26^,^27^,^59^ There are at least 10 different VACV intracellular inhibitors of NF-*κ*B activation^31^–^33^,^60^–^67^ and several other proteins that block IRF3 activation^23^,^65^,^67^–^69^ and therefore it is likely that removal of some of these immunomodulators alone or in combination may improve immunological memory. Hence, these mutant viruses are valuable tools to identify factors promoting immunological memory as well as functioning as improved vaccines.

## Abbreviations

VACV: vaccinia virus

## Supporting Information

**Figure S1.** Viruses with N1 mutants replicate equally *in vivo* early after infection. **Figure S2.** Mutation of vaccinia virus (VACV) N1 does not affect the draining lymph nodes cell numbers during infection. **Figure S3.** Flow cytometric gating strategies.

## References

[b1] Badovinac VP, Harty JT (2006). Programming, demarcating, and manipulating CD8^+^ T-cell memory. Immunol Rev.

[b2] Ahmed R, Gray D (1996). Immunological memory and protective immunity: understanding their relation. Science.

[b3] Pulendran B, Ahmed R (2011). Immunological mechanisms of vaccination. Nat Immunol.

[b4] Volkert M, Marker O, Bro-Jorgensen K (1974). Two populations of T lymphocytes immune to the lymphocytic choriomeningitis virus. J Exp Med.

[b5] Lanzavecchia A, Sallusto F (2005). Understanding the generation and function of memory T cell subsets. Curr Opin Immunol.

[b6] Sallusto F, Lenig D, Forster R, Lipp M, Lanzavecchia A (1999). Two subsets of memory T lymphocytes with distinct homing potentials and effector functions. Nature.

[b7] Zaph C, Uzonna J, Beverley SM, Scott P (2004). Central memory T cells mediate long-term immunity to *Leishmania major* in the absence of persistent parasites. Nat Med.

[b8] Cerwenka A, Morgan TM, Dutton RW (1999). Naive, effector, and memory CD8 T cells in protection against pulmonary influenza virus infection: homing properties rather than initial frequencies are crucial. J Immunol.

[b9] Castiglioni P, de Hall S, Jacovetty EL, Ingulli E, Zanetti M (2008). Protection against influenza A virus by memory CD8 T cells requires reactivation by bone marrow-derived dendritic cells. J Immunol.

[b10] Zanetti M, Franchini G (2006). T cell memory and protective immunity by vaccination: is more better?. Trends Immunol.

[b11] Fenner F, Henderson DA, Arita I, Jezek Z, Ladnyi ID (1988). Smallpox and its eradication.

[b12] Crotty S, Felgner P, Davies H, Glidewell J, Villarreal L, Ahmed R (2003). Long-term B cell memory in humans after smallpox vaccination. J Immunol.

[b13] Hammarlund E, Lewis MW, Hansen SG, Strelow LI, Nelson JA, Sexton GJ, Hanifin JM, Slifka MK (2003). Duration of antiviral immunity after smallpox vaccination. Nat Med.

[b14] Pütz MM, Alberini I, Midgley CM, Manini I, Montomoli E, Smith GL (2005). Prevalence of antibodies to vaccinia virus after smallpox vaccination in Italy. J Gen Virol.

[b15] Taub DD, Ershler WB, Janowski M (2008). Immunity from smallpox vaccine persists for decades: a longitudinal study. Am J Med.

[b16] Moss B (2011). Smallpox vaccines: targets of protective immunity. Immunol Rev.

[b17] Mackett M, Smith GL (1986). Vaccinia virus expression vectors. J Gen Virol.

[b18] Moss B, Flexner C (1987). Vaccinia virus expression vectors. Annu Rev Immunol.

[b19] Goebel SJ, Johnson GP, Perkus ME, Davis SW, Winslow JP, Paoletti E (1990). The complete DNA sequence of vaccinia virus. Virology.

[b20] Smith GL, Benfield CT, Maluquer de Motes C, Mazzon M, Ember SW, Ferguson BJ, Sumner RP (2013). Vaccinia virus immune evasion: mechanisms, virulence and immunogenicity. J Gen Virol.

[b21] Alcami A, Smith GL (1996). A mechanism for the inhibition of fever by a virus. Proc Natl Acad Sci USA.

[b22] Ferguson BJ, Mansur DS, Peters NE, Ren H, Smith GL (2012). DNA-PK is a DNA sensor for IRF-3-dependent innate immunity. Elife.

[b23] Peters NE, Ferguson BJ, Mazzon M (2013). A mechanism for the inhibition of DNA-PK-mediated DNA sensing by vaccinia virus. PLoS Pathog.

[b24] Clark RH, Kenyon JC, Bartlett NW, Tscharke DC, Smith GL (2006). Deletion of gene A41L enhances vaccinia virus immunogenicity and vaccine efficacy. J Gen Virol.

[b25] Staib C, Kisling S, Erfle V, Sutter G (2005). Inactivation of the viral interleukin 1β receptor improves CD8^+^ T-cell memory responses elicited upon immunization with modified vaccinia virus Ankara. J Gen Virol.

[b26] Sumner RP, Ren H, Smith GL (2013). Deletion of immunomodulator C6 from vaccinia virus strain Western Reserve enhances virus immunogenicity and vaccine efficacy. J Gen Virol.

[b27] Garcia-Arriaza J, Najera JL, Gomez CE, Tewabe N, Sorzano CO, Calandra T, Roger T, Esteban M (2011). A candidate HIV/AIDS vaccine (MVA-B) lacking vaccinia virus gene C6L enhances memory HIV-1-specific T-cell responses. PLoS ONE.

[b28] Iwasaki A, Medzhitov R (2010). Regulation of adaptive immunity by the innate immune system. Science.

[b29] Bartlett N, Symons JA, Tscharke DC, Smith GL (2002). The vaccinia virus N1L protein is an intracellular homodimer that promotes virulence. J Gen Virol.

[b30] Antoine G, Scheiflinger F, Dorner F, Falkner FG (1998). The complete genomic sequence of the modified vaccinia Ankara strain: comparison with other orthopoxviruses. Virology.

[b31] DiPerna G, Stack J, Bowie AG (2004). Poxvirus protein N1L targets the I-κB kinase complex, inhibits signaling to NF-κB by the tumor necrosis factor superfamily of receptors, and inhibits NF-κB and IRF3 signaling by toll-like receptors. J Biol Chem.

[b32] Graham SC, Bahar MW, Cooray S (2008). Vaccinia virus proteins A52 and B14 Share a Bcl-2-like fold but have evolved to inhibit NF-κB rather than apoptosis. PLoS Pathog.

[b33] Maluquer de Motes C, Cooray S, Ren H (2011). Inhibition of apoptosis and NF-κB activation by vaccinia protein N1 occur via distinct binding surfaces and make different contributions to virulence. PLoS Pathog.

[b34] Cooray S, Bahar MW, Abrescia NG (2007). Functional and structural studies of the vaccinia virus virulence factor N1 reveal a Bcl-2-like anti-apoptotic protein. J Gen Virol.

[b35] Kotwal GJ, Hugin AW, Moss B (1989). Mapping and insertional mutagenesis of a vaccinia virus gene encoding a 13,800-Da secreted protein. Virology.

[b36] Aoyagi M, Zhai D, Jin C, Aleshin AE, Stec B, Reed JC, Liddington RC (2007). Vaccinia virus N1L protein resembles a B cell lymphoma-2 (Bcl-2) family protein. Protein Sci.

[b37] Tscharke DC, Reading PC, Smith GL (2002). Dermal infection with vaccinia virus reveals roles for virus proteins not seen using other inoculation routes. J Gen Virol.

[b38] Tscharke DC, Smith GL (1999). A model for vaccinia virus pathogenesis and immunity based on intradermal injection of mouse ear pinnae. J Gen Virol.

[b39] Reading PC, Smith GL (2003). Vaccinia virus interleukin-18-binding protein promotes virulence by reducing γ interferon production and natural killer and T-cell activity. J Virol.

[b40] Ren H, Shen J, Tomiyama-Miyaji C, Watanabe M, Kainuma E, Inoue M, Kuwano Y, Abo T (2006). Augmentation of innate immunity by low-dose irradiation. Cell Immunol.

[b41] Yuen TJ, Flesch IE, Hollett NA, Dobson BM, Russell TA, Fahrer AM, Tscharke DC (2010). Analysis of A47, an immunoprevalent protein of vaccinia virus, leads to a reevaluation of the total antiviral CD8^+^ T cell response. J Virol.

[b42] Zhang WH, Wilcock D, Smith GL (2000). Vaccinia virus F12L protein is required for actin tail formation, normal plaque size, and virulence. J Virol.

[b43] Putz MM, Midgley CM, Law M, Smith GL (2006). Quantification of antibody responses against multiple antigens of the two infectious forms of Vaccinia virus provides a benchmark for smallpox vaccination. Nat Med.

[b44] Alcami A, Smith GL (1995). Vaccinia, cowpox, and camelpox viruses encode soluble γ interferon receptors with novel broad species specificity. J Virol.

[b45] Tscharke DC, Karupiah G, Zhou J (2005). Identification of poxvirus CD8^+^ T cell determinants to enable rational design and characterization of smallpox vaccines. J Exp Med.

[b46] Law M, Putz MM, Smith GL (2005). An investigation of the therapeutic value of vaccinia-immune IgG in a mouse pneumonia model. J Gen Virol.

[b47] Jacobs N, Bartlett NW, Clark RH, Smith GL (2008). Vaccinia virus lacking the Bcl-2-like protein N1 induces a stronger natural killer cell response to infection. J Gen Virol.

[b48] Gratz MS, Suezer Y, Kremer M (2011). N1L is an ectromelia virus virulence factor and essential for *in vivo* spread upon respiratory infection. J Virol.

[b49] Mathew A, O'Bryan J, Marshall W, Kotwal GJ, Terajima M, Green S, Rothman AL, Ennis FA (2008). Robust intrapulmonary CD8 T cell responses and protection with an attenuated N1L deleted vaccinia virus. PLoS ONE.

[b50] Kaech SM, Cui W (2012). Transcriptional control of effector and memory CD8^+^ T cell differentiation. Nat Rev Immunol.

[b51] Sun JC, Madera S, Bezman NA, Beilke JN, Kaplan MH, Lanier LL (2012). Proinflammatory cytokine signaling required for the generation of natural killer cell memory. J Exp Med.

[b52] Plumlee CR, Sheridan BS, Cicek BB, Lefrancois L (2013). Environmental cues dictate the fate of individual CD8^+^ T cells responding to infection. Immunity.

[b53] Tan JT, Ernst B, Kieper WC, LeRoy E, Sprent J, Surh CD (2002). Interleukin (IL)-15 and IL-7 jointly regulate homeostatic proliferation of memory phenotype CD8^+^ cells but are not required for memory phenotype CD4^+^ cells. J Exp Med.

[b54] Pahl HL (1999). Activators and target genes of Rel/NF-κB transcription factors. Oncogene.

[b55] Memet S, Laouini D, Epinat JC (1999). IκBε-deficient mice: reduction of one T cell precursor subspecies and enhanced Ig isotype switching and cytokine synthesis. J Immunol.

[b56] Ishikawa H, Claudio E, Dambach D, Raventos-Suarez C, Ryan C, Bravo R (1998). Chronic inflammation and susceptibility to bacterial infections in mice lacking the polypeptide (p)105 precursor (NF-κB1) but expressing p50. J Exp Med.

[b57] Caamano JH, Rizzo CA, Durham SK, Barton DS, Raventos-Suarez C, Snapper CM, Bravo R (1998). Nuclear factor (NF)-κB2 (p100/p52) is required for normal splenic microarchitecture and B cell-mediated immune responses. J Exp Med.

[b58] Coffman RL, Sher A, Seder RA (2010). Vaccine adjuvants: putting innate immunity to work. Immunity.

[b59] Garcia-Arriaza J, Arnaez P, Gomez CE, Sorzano CO, Esteban M (2013). Improving adaptive and memory immune responses of an HIV/AIDS vaccine candidate MVA-B by deletion of vaccinia virus genes (C6L and K7R) blocking interferon signaling pathways. PLoS One.

[b60] Chen RA, Ryzhakov G, Cooray S, Randow F, Smith GL (2008). Inhibition of IκB kinase by vaccinia virus virulence factor B14. PLoS Pathog.

[b61] Ember SW, Ren H, Ferguson BJ, Smith GL (2012). Vaccinia virus protein C4 inhibits NF-κB activation and promotes virus virulence. J Gen Virol.

[b62] Gedey R, Jin XL, Hinthong O, Shisler JL (2006). Poxviral regulation of the host NF-κB response: the vaccinia virus M2L protein inhibits induction of NF-κB activation via an ERK2 pathway in virus-infected human embryonic kidney cells. J Virol.

[b63] Harte MT, Haga IR, Maloney G (2003). The poxvirus protein A52R targets Toll-like receptor signaling complexes to suppress host defense. J Exp Med.

[b64] Mansur DS, Maluquer de Motes C, Unterholzner L (2013). Poxvirus targeting of E3 ligase beta-TrCP by molecular mimicry: a mechanism to inhibit NF-κB activation and promote immune evasion and virulence. PLoS Pathog.

[b65] Schroder M, Baran M, Bowie AG (2008). Viral targeting of DEAD box protein 3 reveals its role in TBK1/IKKε-mediated IRF activation. EMBO J.

[b66] Shisler JL, Jin XL (2004). The vaccinia virus K1L gene product inhibits host NF-κB activation by preventing IκBα degradation. J Virol.

[b67] Stack J, Haga IR, Schroder M (2005). Vaccinia virus protein A46R targets multiple Toll-like-interleukin-1 receptor adaptors and contributes to virulence. J Exp Med.

[b68] Ferguson BJ, Benfield CT, Ren H, Lee VH, Frazer GL, Strnadova P, Sumner RP, Smith GL (2013). Vaccinia virus protein N2 is a nuclear IRF3 inhibitor that promotes virulence. J Gen Virol.

[b69] Unterholzner L, Sumner RP, Baran M (2011). Vaccinia virus protein C6 is a virulence factor that binds TBK-1 adaptor proteins and inhibits activation of IRF3 and IRF7. PLoS Pathog.

