# Supplementary material for: Enhancement of CD8+ T-cell memory by removal of a vaccinia virus nuclear factor-κB inhibitor
Source: Immunology. 2015 Apr 14;145(1):34–49. doi: 10.1111/imm.12422 (PMC4405322; doi:10.1111/imm.12422)
Supplement: Supplementary file 1 [file imm0145-0034-sd1.pptx]

## Slide 1
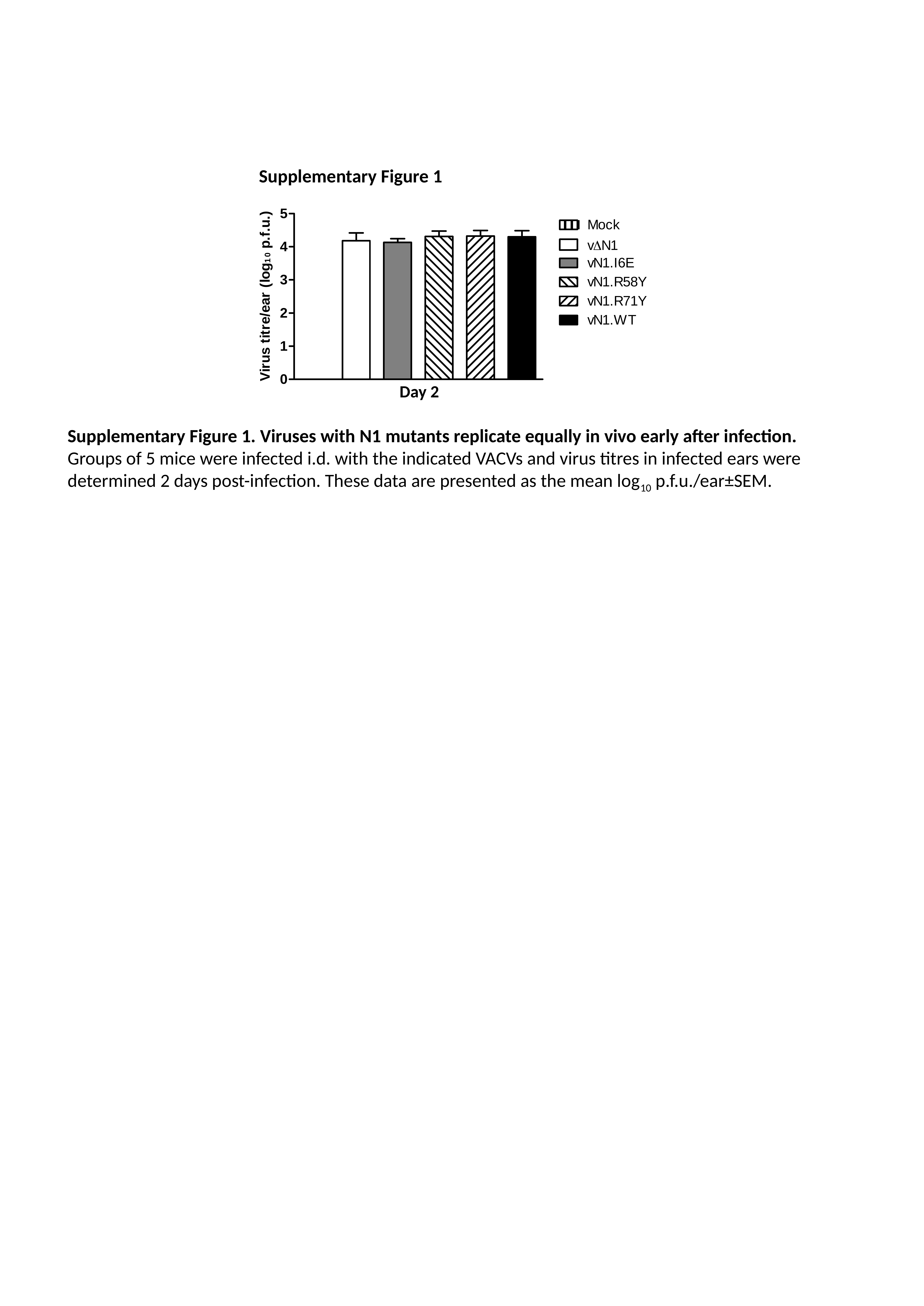

Supplementary Figure 1
Day 2
Supplementary Figure 1. Viruses with N1 mutants replicate equally in vivo early after infection.
Groups of 5 mice were infected i.d. with the indicated VACVs and virus titres in infected ears were
determined 2 days post-infection. These data are presented as the mean log10 p.f.u./ear±SEM.

## Slide 2
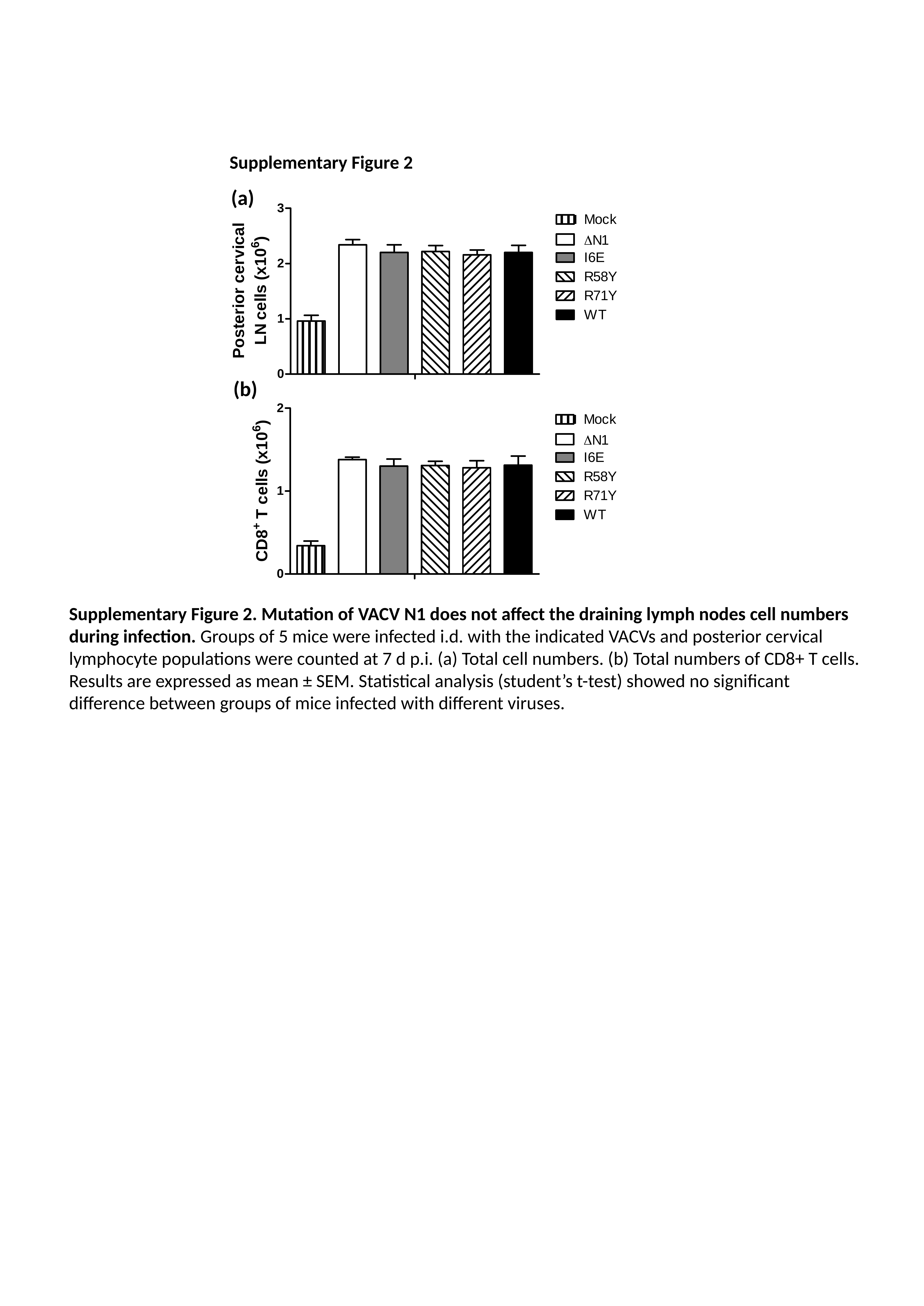

Supplementary Figure 2
(a)
(b)
Supplementary Figure 2. Mutation of VACV N1 does not affect the draining lymph nodes cell numbers
during infection. Groups of 5 mice were infected i.d. with the indicated VACVs and posterior cervical
lymphocyte populations were counted at 7 d p.i. (a) Total cell numbers. (b) Total numbers of CD8+ T cells.
Results are expressed as mean ± SEM. Statistical analysis (student’s t-test) showed no significant
difference between groups of mice infected with different viruses.

## Slide 3
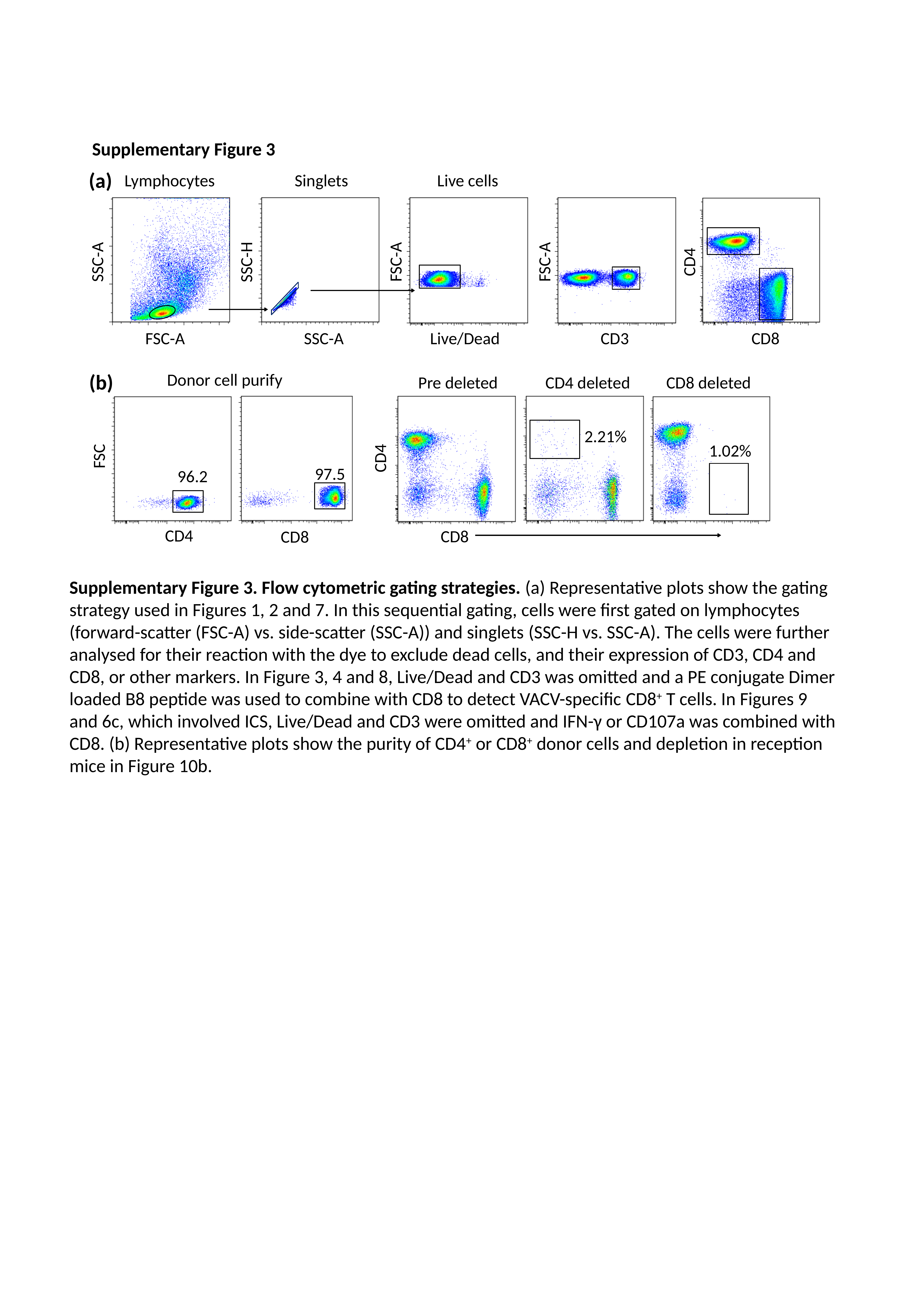

Supplementary Figure 3
Lymphocytes
Singlets
Live cells
(a)
SSC-A
SSC-H
FSC-A
FSC-A
CD4
FSC-A
SSC-A
Live/Dead
CD3
CD8
Pre deleted
CD4 deleted
CD8 deleted
(b)
Donor cell purify
2.21%
1.02%
FSC
CD4
97.5
96.2
CD8
CD4
CD8
Supplementary Figure 3. Flow cytometric gating strategies. (a) Representative plots show the gating
strategy used in Figures 1, 2 and 7. In this sequential gating, cells were first gated on lymphocytes
(forward-scatter (FSC-A) vs. side-scatter (SSC-A)) and singlets (SSC-H vs. SSC-A). The cells were further
analysed for their reaction with the dye to exclude dead cells, and their expression of CD3, CD4 and
CD8, or other markers. In Figure 3, 4 and 8, Live/Dead and CD3 was omitted and a PE conjugate Dimer
loaded B8 peptide was used to combine with CD8 to detect VACV-specific CD8+ T cells. In Figures 9
and 6c, which involved ICS, Live/Dead and CD3 were omitted and IFN-γ or CD107a was combined with
CD8. (b) Representative plots show the purity of CD4+ or CD8+ donor cells and depletion in reception
mice in Figure 10b.
